# Superior Sagittal Venous Sinus Thrombosis in a Patient with Illicit Testosterone Use

**DOI:** 10.7759/cureus.5491

**Published:** 2019-08-26

**Authors:** Ariba Hashmi, Paul Kim, Syed W Ahmad, Jason Faucheux, Naz Gandikal

**Affiliations:** 1 Medical Education, Nova Southeastern University, Dr. Kiran C. Patel College of Osteopathic Medicine, Fort Lauderdale, USA; 2 Family Medicine, Advent Health Orlando, Orlando, USA

**Keywords:** superior sagittal sinus, cerebral venous sinus thrombosis, cord sign, delta sign, testosterone replacement therapy, androgenic anabolic steroids, anabolic steroids, coagulation, general tonic-clonic seizures, hypercoagulability

## Abstract

Cerebral venous sinus thrombosis is a challenging diagnosis due in part to its variable clinical presentation and rarity. The annual incidence ranges from 0.22 to 1.57 per 100,000. The etiology of such disease is related to hypercoagulability states. Although illicit androgen use is a well-known cause of prothrombotic states, its risk of causing cerebral venous sinus thrombosis has been infrequently reported. We present the case of a 33-year-old male with no known past medical history who presented to the emergency department (ED) with persistent seizure activity, neurological deficits, and history of worsening headaches who was found to have an extensive superior sagittal sinus thrombosis on imaging. Radiologic findings demonstrated pathognomonic findings of cord sign and delta sign, the previous being highly specific but of low incidence. An inconclusive hypercoagulability workup prompted further questioning which revealed illicit androgenic anabolic steroid use. Prompt treatment with anticoagulation and anti-seizure medication was pursued with full resolution of his neurologic symptomatology.

## Introduction

Cerebral venous sinus thrombosis (CVST) is an uncommon cause of cerebrovascular pathology relative to arterial strokes. The annual incidence ranges from 0.22 to 1.57 per 100,000 [1]. Patients may present with varying degrees of nonspecific neurologic symptomatology making the diagnosis difficult and requiring a high index of suspicion. Symptoms can manifest as a result of ischemia, infarction, or hemorrhage with focal cerebral injury. In addition, venous thrombosis reduces drainage of the cerebral microvasculature, leading to an increase in upstream intracranial pressure and further decline of normal cerebral hemodynamics [2]. Certain hypercoagulable states and risk factors, such as pregnancy, have been associated with CVST but androgenic anabolic steroid (AAS) use is relatively rare.

## Case presentation

**Figure 1:**
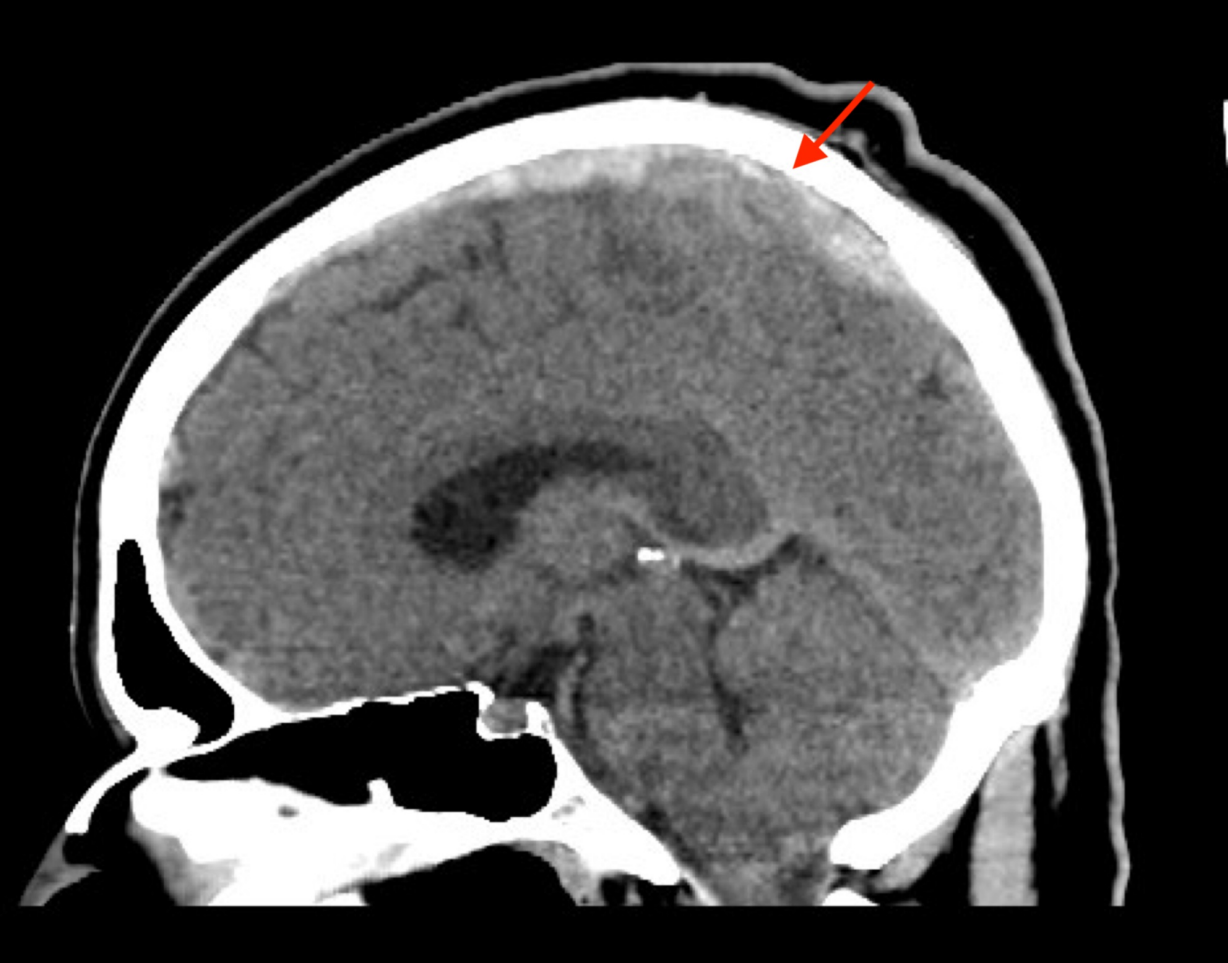
Sagittal non-contrast computed tomography (CT) of the thrombus demonstrating a cord sign

We report the case of a 33-year-old Hispanic male who was brought to the emergency department (ED) by emergency medical service (EMS) after his wife witnessed an episode of sustained tonic-clonic seizure activity while at home. Several weeks prior, the patient was seen in the ED by a mid-level provider for non-specific headaches for which a non-contrast computed tomography (CT) scan of the head was ordered with no dictated significant findings. With a negative workup, the patient was discharged home with muscle relaxers for a presumed tension headache. On readmission to the ED, a stat repeat non-contrast CT head was ordered due to new-onset sustained seizure activity. Sagittal imaging revealed a positive cord sign with hyperdensity of the superior sagittal sinus (Figure 1). 

**Figure 2:**
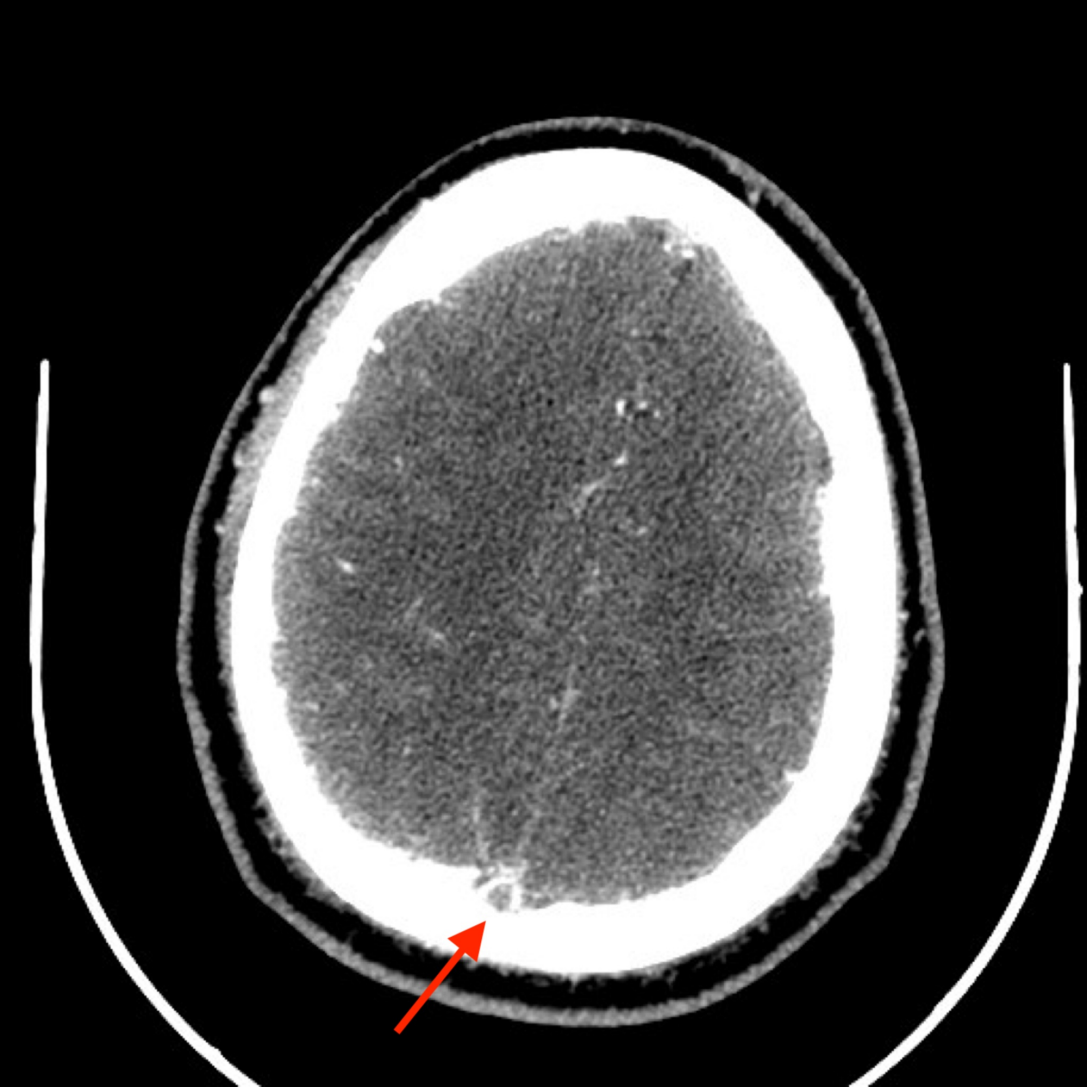
Axial computed tomography venography (CTV) of the thrombus demonstrating delta sign

This suggested an extensive superior sagittal sinus thrombosis extending the entire length of the sinus. Axial CT venography of the brain revealed an empty delta sign consisting of a triangular area of contrast enhancement with a center of low-attenuation revealing the thrombus (Figure 2).

**Figure 3:**
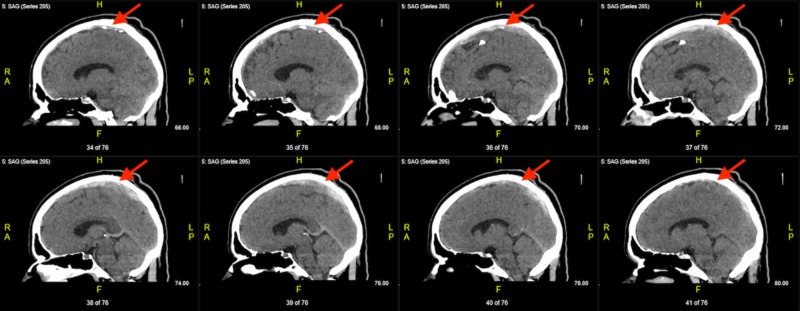
Serial sagittal non-contrast computed tomography (CT) demonstrating a left-to-right progression of sinuses with thrombus

The large burden of the thrombosis extending from the frontal end of the superior sagittal sinus and ending at the confluence of the sinuses can be visualized in the serial sagittal images (Figure 3).

Due to persistent episodes of seizure activity, the patient was intubated and given a loading dose of Dilantin® (phenytoin) and Keppra® (levetiracetam). Weight-based Lovenox® (enoxaparin) was started for anticoagulation, and the patient was transferred to the intensive care unit (ICU). Further investigation to reveal the etiology of the thrombosis was pursued. Hypercoagulability studies showed non-specific findings but failed to clarify a true etiology (Table 1). Further questioning with the patient's spouse and family revealed that the patient had recently started receiving 300 mg/ml of AAS injections from his personal fitness trainer for approximately the last 45 days. Within the month, the patient started complaining of dizziness, weakness, and near syncopal episodes. Worsening headaches prompted his initial visit to the ED. Per the patient's spouse, she noted his gait to be unsteady and that the patient was utilizing his right-sided extremities while neglecting his left side. Of note, the patient had been diagnosed with low testosterone levels by his primary care provider prior to receiving AAS injections. He had been receiving prescribed testosterone replacement therapy until his insurance refused coverage, prompting the patient to seek AAS from his personal trainer. Androgenic studies were pursued, and the results highlighted abnormally high testosterone levels, offering a source of etiology for the patient’s venous sinus thrombosis formation (Table 2). As the patient stabilized and was extubated, the neurological examination found him to have left-sided hemineglect. The patient was otherwise neurologically intact with no other gross deficits. He did incidentally have concerns for aspiration pneumonia but was appropriately treated with antibiotics. With continued observation and treatment, a complete resolution of his neurologic deficits was achieved, and intravenous (IV) anticoagulation was bridged to oral warfarin. The patient was discharged home with oral anticoagulation, prophylactic anti-seizure medications, and the remaining doses of antibiotics for aspiration pneumonia. He was strongly advised to discontinue all testosterone supplementation until further outpatient follow-up.

**Table 1:** Hypercoagulability Testing Results

Significant Coagulability Testing	Levels
D-Dimer	0.9 (0.0 - 0.5)
Protein S Activity	> 150
Protein C Activity	> 150
Antithrombin III	150
Factor 8	> 500
Factor 2 Gene Mutation	Negative
Factor V Leiden Mutation	Negative

**Table 2:** Androgen Testing Results

Androgen Testing	Levels
Testosterone Level	968 ng/dl (300 - 950 ng/dl)
Testosterone-free Adult Male	334 pg/mL (47 - 244 pg/mL)
% Free Testosterone	3.5% (1.6 - 2.9%)
Bioavailable Testosterone	891 ng/dL (72.0 - 235.0 ng/dL)
Sex Hormone Binding Globulin	5 nmol/L (11 - 80 nmol/L)

## Discussion

Dural venous sinuses are found in the superficial meningeal layer where they receive blood from the cerebral veins and cerebrospinal fluid (CSF) from the arachnoid granulations. The sinuses collectively empty into the internal jugular vein. Therefore, thrombosis of the dural sinus may attribute symptoms to a lack of venous outflow, as well as increased intracranial pressure. The thrombosis can eventually lead to hemorrhage due to the lack of drainage and cerebral parenchymal injury. CVST accounts for less than 1% of all strokes, with a median age of 37 years, affecting women more commonly than men at a 3:1 ratio [3]. The case presented highlights a male individual less than the expected age. The patient’s symptoms of headache, dizziness, seizure activity, and hemineglect have previously been noted to occur in patients with isolated sinus thrombosis compared to other dural sinus regions [4]. In a previous cohort study, 40% of individuals studied with CVST were found to have seizure activity when presenting (a far greater incidence when compared to arterial cerebrovascular strokes). Focal deficits in the absence of focal injury have also been reported in as many as 37% of cases, as seen in this case [5]. The extent of the patient’s thrombosis was also of significance as it spanned the length of the entire superior sagittal sinus attributing to the positive radiologic cord sign, a relatively rare image finding compared to the delta sign which was also visualized [6]. The patient’s symptoms and radiologic findings support the importance of considering seizures, focal neurological deficit, and the radiologic cord sign when making an accurate diagnosis of cerebral vein thrombosis.

The etiology of sinus thrombosis is most often associated with prothrombotic states. AAS use is associated with thrombus formation through several proposed mechanisms. The effect of AAS use on lipid metabolism has been well-documented. A literature review which quantified the effects of AAS use on serum lipid levels was published in the Archives of Internal Medicine in 1991 and showed that AAS use reduced high-density lipoprotein (HDL) levels by 52% and increased low-density lipoprotein (LDL) levels by 36% [7]. AAS use has also been shown to induce myocardial hypertrophy and directly inhibit myocardial contractility and relaxation, leading to pro-arrhythmic states [8]. Furthermore, AAS use has been shown to directly affect hemostasis. A study by Ferenchick et al. examined the relationship between AAS use and coagulation. The study compared 49 weightlifters separated into AAS users and non-users. Plasma assays showed a 10% increase in thrombin/antithrombin complexes, a 20% increase in prothrombin fragment 1+2, and a 9% increase in D-dimer levels in the AAS users. The activities of antithrombin III and protein S were also higher in AAS users by 18% and 19%, respectively. The study concluded that AAS use increased activation of both the coagulation and fibrinolytic pathways, leading to an abnormal hemostatic response which could potentiate thrombus formation [9]. These findings offer some perspective into the non-specific coagulation study results seen in the case presented (Table 1). In addition, AAS use has demonstrated inhibition of vasodilation from endothelial-dependent and independent mechanisms, causing further homeostatic insult in animal models [10].

Comparatively, the metabolic effects of testosterone replacement therapy do not induce the same prothrombotic changes as does AAS use. A study by Stefen et al. examined the effects of long-term testosterone replacement in hypogonadal and elderly men on lipid and lipoprotein levels. Twenty-two subjects were enrolled in the study, split between 11 male subjects with hypopituitarism and 11 otherwise healthy elderly males. Results specific for the hypogonadal males showed a decrease in total cholesterol from 255 ± 12.1 mg/dl to 214 ± 10.6 mg/dl after six months and a further decrease to 206 ± 9 mg/dl after one year of treatment with a P-value < 0.0001. Similarly, the LDL concentrations decreased from 178 ± 10.3 mg/dl to 149 ± 10.2 mg/dl after six months and 140 ± 7.3 mg/dl after one year of treatment with a P-value < 0.001 [11].

Furthermore, a literature review which quantified the cardiovascular effects related to hypogonadism and testosterone therapy was published in the American Journal of Cardiology in 2005. Observational studies, interventional studies, and short-term data were included in the review. Short-term testosterone therapy was determined to be beneficial and lower cardiovascular risk factors. Among the effects, the most prominent were improved insulin sensitivity, central obesity, decreased total cholesterol, and LDL. Some studies showed testosterone therapy had an HDL-lowering effect but was found to be insignificant in other studies. The long-term cardiovascular effects were determined to be neutral to beneficial. In addition, testosterone therapy was not contraindicated in men with cerebrovascular disease and hypogonadism [12].

Another contributing factor to consider is the recurrent transient decreases in cerebral blood flow. In a study by Neerav et al., deep inspiratory breath-holding, as in the Valsalva maneuver, was shown to reduce venous blood flow in the superior sagittal sinus by up to 30.8%. Specific to our patient, frequent resistance training and use of the Valsalva maneuver might have had an impact in thrombogenesis [13].

The patient presented in this case could have developed a CVST through a combination of abnormal hemostasis, increased platelet aggregation, elevated factor VIII levels, and transient decreases in cerebral blood flow.

## Conclusions

CVST is a clinically challenging diagnosis due to its variable clinical presentation. This case highlights the importance of several key factors that culminated in the patient’s acute presentation. The primary care physician of this patient prescribed testosterone replacement based off of current recommendation guidelines, and it is assumed that the patient was informed of the potential side effects. However, this case emphasizes the importance of active patient education on pursued treatment modality, as well as the ramifications of departure from medical advice. Unknowingly, the patient presumed both prescription hormone replacement and AAS were analogous, which further exemplifies the need for patient education regarding hormone replacement therapy. In addition, a more thorough history would have revealed the patient’s use of AAS during the first ED admission. This information, as part of the social and medical history, along with the nonspecific neurologic symptoms, would have raised the index of suspicion of CVST for both the provider and radiologist, emphasizing the importance of a thorough history. Although the incidence of cerebrovascular accidents attributable to CVST is low, it is nevertheless an important component of the evaluation of a patient with nonspecific neurologic symptomatology.
